# The concept of ‘vulnerability’ in research ethics: an in-depth analysis of policies and guidelines

**DOI:** 10.1186/s12961-016-0164-6

**Published:** 2017-02-07

**Authors:** Dearbhail Bracken-Roche, Emily Bell, Mary Ellen Macdonald, Eric Racine

**Affiliations:** 1 Neuroethics Research Unit, Institut de recherches cliniques de Montréal, 110 Avenue des Pins Ouest, Montréal, QC H2W 1R7 Canada; 2 Biomedical Ethics Unit and Division of Experimental Medicine, McGill University, Montréal, QC Canada; 3 Faculty of Dentistry, Oral Health and Society Research Unit, McGill University, 2001 McGill College, Suite 500, Montréal, QC H3A 1G1 Canada; 4 Department of Medicine and Department of Social and Preventive Medicine, Université de Montréal, Montréal, QC Canada; 5 Department of Neurology and Neurosurgery, McGill University, Montréal, QC Canada

**Keywords:** Research ethics, Vulnerable populations, Vulnerability, Research policy, Ethics policy, Research oversight

## Abstract

**Background:**

The concept of vulnerability has held a central place in research ethics guidance since its introduction in the United States Belmont Report in 1979. It signals mindfulness for researchers and research ethics boards to the possibility that some participants may be at higher risk of harm or wrong. Despite its important intended purpose and widespread use, there is considerable disagreement in the scholarly literature about the meaning and delineation of vulnerability, stemming from a perceived lack of guidance within research ethics standards. The aim of this study was to assess the concept of vulnerability as it is employed in major national and international research ethics policies and guidelines.

**Methods:**

We conducted an in-depth analysis of 11 (five national and six international) research ethics policies and guidelines, exploring their discussions of the definition, application, normative justification and implications of vulnerability.

**Results:**

Few policies and guidelines explicitly defined vulnerability, instead relying on implicit assumptions and the delineation of vulnerable groups and sources of vulnerability. On the whole, we found considerable richness in the content on vulnerability across policies, but note that this relies heavily on the structure imposed on the data through our analysis.

**Conclusions:**

Our results underscore a need for policymakers to revisit the guidance on vulnerability in research ethics, and we propose that a process of stakeholder engagement would well-support this effort.

## Background: the function of vulnerability in research ethics guidance and policy

Research on human subjects is thought to be fundamentally ethically challenging, requiring ethics standards to guide researchers as well as approval and oversight of research proposals from independent committees. Society allows researchers to invite individuals to participate in research once certain conditions are met, including a research ethics board’s (REB, also known as Institutional Review Boards, or IRBs, or Research Ethics Committees, or RECs) determination that risks and benefits are appropriately balanced, that the proposed strategy for subject recruitment is fair, and that voluntary, informed consent will be sought from each potential subject [1]. The concept of vulnerability, which finds it origins in the United States Belmont Report of 1979 [2], plays a central role in research ethics thinking, drawing attention to situations where these conditions may not be met [1]. Since 1979, the number of legal and non-legal research ethics policies and guidelines has increased tremendously and, with them, the use and scope of the concept of vulnerability or vulnerable populations [2, 3]. However, there is much scholarly disagreement over the appropriate meaning and application of this concept in research ethics, and policymakers are charged with the challenge of navigating this contentious landscape in the development and refinement of research guidelines and policies [4]. A growing body of literature critiques and aims to advance the way vulnerability is conceptualised and employed in research ethics, with major debates regarding foundational elements of this important ethical concept [5–10].

### Major debates surrounding the concept of vulnerability in research ethics

There is widespread agreement that some research participants may be particularly vulnerable and in need of special protections, yet the concept of vulnerability itself has been described as ‘vague’ [11], with a lack of consensus in the scholarly literature regarding the concept’s central features. Contrasting accounts have been proposed regarding the justification of vulnerability and which ethical principles translate into obligations for the special protection of vulnerable research participants. Some accounts propose a justice-based reason for protection, concerned with the fairness of participant recruitment and of the distribution of research burdens and benefits [12, 13]. Others ground vulnerability in a principle of autonomy or respect for persons, suggesting that persons who cannot provide informed and voluntary consent are susceptible to harm because they are not able to protect their interests [5, 13]. These approaches are not mutually exclusive, but we must at least be able to identify which ethical principles define to whom we owe special consideration. In research, there are often gaps between the rules intended to govern and the practices at hand, requiring those tasked with the implementation of these rules to interpret and apply them in their specific context [3]. An ethical foundation is needed for these interpretations; otherwise, it is difficult to understand the intentions of the authoring parties or apply the rule to the situation at hand [3]. In this context, a better understanding of the justifications of vulnerability becomes a crucial goal of scholarly work in this area.

The application of vulnerability and its scope in research has also been a subject of much debate. In particular, vulnerability has been charged with being both too broad and too narrow. An overly broad concept captures all research participants, creating conceptual confusion over the meaning of ‘special protections’, while an overly narrow concept may leave some vulnerable participants at risk and without the needed protection [5, 11, 12, 14]. Practically, a definition of vulnerability must be comprehensive enough to capture those in need of additional protections without overburdening participants for whom protection beyond the norm is unnecessary. Further, it must provide researchers and research ethics boards with the information necessary to identify those who are vulnerable, as well as what they might be vulnerable to. There are compelling arguments against narrow definitions of vulnerable groups that support the identification of specific factors within the research context and the participants’ personal situation that create possible vulnerabilities [6].

The foundational debate about the concept of vulnerability revolves around its definition, with various proposals made for its delineation in the literature. Arguing that vulnerability lacks an organising principle, Hurst [5] suggests that vulnerable persons are properly conceived of as those who have “*an identifiably increased likelihood of incurring additional or greater wrong*”. This account emphasises that both individual and situational factors must be evaluated in defining vulnerability because being overly focused on individual characteristics can obscure features of the research protocol or environment that may harm participants. Luna [6] argues that vulnerability must be conceived of as ‘relational’, in that vulnerabilities can only be discovered by examining an individual in context, and ‘dynamic’, since one’s vulnerability depends on one’s context. Luna and Vanderpoel [14] describe layers of vulnerability which arise from interactions between an individual’s characteristics and their environment, and which interact with one another to create an inextricably context-dependent vulnerability.

To our knowledge, an in-depth analysis of the concept of vulnerability as it exists in the policies and guidelines that govern research on human subjects has not been conducted. This is an important gap because, at present, the scholarly literature seeks to advance the concept without an understanding of the full scope of the regulatory context. Without a clear understanding of the conceptualisation and operationalisation of vulnerability in current research ethics, recommendations for its refinement risk being disconnected from the range of policy options. To explore the diversity of options with respect to the enshrinement and application of the concept of vulnerability in research ethics guidelines, we conducted an in-depth analysis of major national and international research ethics policies and guidelines.

## Methods

### Sampling

Inspired by previous research ethics policy analyses [3, 15], we compiled a sample of internationally- and nationally-adopted research ethics guidelines and policies, focusing on Canada (the authors’ own regulatory context) and regions with similar demographic and legal structures to Canada, including Australia, the European Union, the United Kingdom, and the United States [15]. We began our search using a compilation of international human research standards produced by the Office for Human Research Protections of the United States Department of Health and Human Services [16]. Additionally, we performed secondary searches of the references of any included guidelines and policies for relevant, non-duplicated documents.

Our primary goal was to build a sample of guidelines and policies that discussed or referenced vulnerability in general health research. As such, we excluded those in which vulnerability was not explicitly discussed (e.g. the Nuremberg code) as well as those focused on specific areas of or issues within research (e.g. paediatric research, genetic research), put forward by professional organisations, or published as working papers, drafts, commentaries, or otherwise less broadly adopted documents. Our final sample included 11 guidelines and policies, six of which are internationally-adopted (i.e. across multiple countries) [17–22] and five of which are nationally-adopted (i.e. within individual countries) [23–27]. All documents were downloaded and saved for data extraction. See Table 1 for an overview of our sample and the key characteristics of included policies.

**Table 1:** Key characteristics of guideline and policy sample

Guideline/Policy	Date	Adopted	Abbreviation	Status	Intended Users	Guiding Ethical Framework/Principles
Declaration of Helsinki	2013	Intl	Declaration of Helsinki	A statement of ethical principles proposing how physicians should act in research; not legally binding	Primarily physicians; others involved in medical research with human subjects are encouraged to adopt its principles	Articles of the Declaration itself are intended as guiding ethical principles for research
Council for International Organizations of Medical Sciences, *International Ethical Guidelines for Biomedical Research Involving Human Subjects*	2002	Intl	CIOMS	A guidance document intended to guide the effective application of the Declaration of Helsinki’s ethical principles in research, especially in low-resource countries; not legally binding	CIOMS member bodies, which include international and national biomedical organisations (e.g. World Medical Association)	Cites three guiding ethical principles: respect for persons, beneficence, and justice
UNESCO Universal Declaration on Bioethics and Human Rights	2005	Intl	UNESCO Declaration	A universal framework of principles to guide States in formulating legislation and policies, as well as to guide the actions of individuals, groups, communities, institutions and corporations, public and private	Addressed to States, but also provides guidance for individuals, groups, communities and corporations, public and private	Articles of the UNESCO Declaration itself are intended as guiding bioethical principles
Directive of 4 April 2001 N°2001/20/EC	2001	EU	EU Clinical Trials Directive	A legislative act that establishes specific provisions for good clinical practice in clinical trials; EU Member States must meet these provisions though the Directive does not legislate how	EU member states	Not explicitly provided; states that “[t]*he accepted basis for the conduct of clinical trials in humans is founded in the protection of human rights and the dignity of the human being… as for instance reflected in the 1996 version of the Helsinki Declaration*” [20]
Regulation of 16 April 2014 N°536/2014	2014	EU	EU Clinical Trials Regulation	A binding legislative act applying to all clinical trials conducted in the EU	EU member states	Not explicitly provided
International Conference on Harmonisation, Good Clinical Practice	1996	US, EU, JP, AUS, CA	ICH GCP	An ethical and scientific quality standard for designing, conducting, recording, and reporting human subject research trials; serves as a unified standard for CA, the EU, JPN, and US to facilitate the mutual acceptance of clinical data by the regulatory authorities in these jurisdictions	Targeted at those involved in the generation of clinical trial data intended to be submitted to regulatory authorities, especially in CA, the EU, JPN, and US; can also be used by others involved in clinical investigations “*that may have an impact of the safety and well-being of human subjects*” [22]	Not explicitly provided; states that “*clinical trials should be conducted in according with the ethical principles that have their origin in the Declaration of Helsinki*” ([22], Art. 2.1)
National Statement on Ethical Conduct in Human Research	2007	AUS	Australian National Statement	Must be used to inform the design, ethical review, and conduct of human research funded by or taking place under the auspices of the bodies that have developed the Statement (i.e. National Health and Medical Research Council, Australian Research Council, Australian Vice-Chancellors’ Committee)	Researchers, members of ethical review bodies, and those involved in research governance, as well as potential research participants	Describes four guiding values and principles: research merit and integrity, justice, beneficence, and respect
Tri-Council Policy Statement, 2nd edition	2014	CA	TCPS2	To be eligible to receive and administer research funds from the federal research agencies responsible for this policy (i.e. Canadian Institutes of Health Research, Natural Sciences and Engineering Research Council of Canada, Social Sciences and Humanities Research Council), institutions must agree to comply with it; while not required to do so, other organisations and entities are encouraged to adopt this Policy to guide the ethical aspects of the design, review and conduct of research involving humans	All those involved in the conduct and review of research funded by the federal research agencies, e.g. institutions, researchers, ethics review boards, etc.	Sets out three core principles: respect for persons, concern for welfare, and justice
Research Governance Framework for Health and Social Care, 2nd edition	2005	UK	UK Research Governance Framework	Sets out a framework of principles, requirements, and standards for the governance of research in health and social care and applies to all research relating to the responsibilities of the Secretary of State for Health	Intended for all those who design research studies, participate in research, host research in their organisation, fund research proposals or infrastructure, manage research, and undertake research	Not explicitly provided
The Belmont Report	1979	US	Belmont Report	A statement of basic ethical principles and guidelines intended to assist in resolving the ethical problems that surround the conduct of research created by the National Commission for the Protection of Human Subjects of Biomedical and Behavioral Research for the Department of Health, Education, and Welfare	Those involved in the review and conduct of research	Lays out three basic ethical principles: respect for persons, beneficence, and justice
Title 45 Code of Federal Regulations, Part 46	1991	US	Common Rule	Serves as a federal policy for human subjects research, and applies to all research conducted or supported by or affiliated with the federal agencies by which is has been adopted	Those involved in the review and conduct of research associated with the federal agencies by which the Common Rule has been adopted	Not explicitly provided, but the Regulations were created on the basis of the Belmont Report

*Intl* international, *EU* European Union, *AUS* Australia, *CA* Canada, *JP* Japan, *UK* United Kingdom, *US* United States

### Inter-policy component analysis

This stage of analysis consisted of an inter-policy analysis, allowing us to capture and explore patterns in the data across our sample. Given our specific interest in understanding how guidelines and policies employ the concept of vulnerability, each document was word-searched for the term ‘vulnerab’. Using this truncated keyword allowed us to identify all uses of the terms ‘vulnerability’ or ‘vulnerable’. We read the broader sections of text surrounding the key terms to facilitate a contextual understanding of how the notion of vulnerability was used.

We employed a content analysis strategy, developing an initial coding guide deductively and refining it inductively. We hypothesised, based on the literature (as described in the Background section), that research ethics guidance on vulnerability should include at least the following basic content: (1) a definition of vulnerability, (2) a discussion of the sources or circumstances from which vulnerability can arise and/or identification of groups likely to be in those circumstances, (3) an explanation of the ethical justification of the concept to aid in its application. A preliminary coding guide was created to capture these content areas. This preliminary guide was applied to a subset of the sample (n = 3). Through this ‘piloting’ stage, the coding scheme was refined inductively by three authors (DBR, EB, ER) to ensure other major areas of content were captured. This resulted in the addition of a fourth content category, ‘implications of vulnerability’, which captures responses to vulnerable participants laid out within the guidelines and policies. Definitions and rules for the application of each code were developed to ensure rigor and thoroughness. Throughout the coding process, three authors (DBR, EB, ER) engaged in open discussions in order to account for any biases of the primary coder (DBR) and to ensure the full depth of the data would be represented through this analytic strategy. Once final coding was complete, it was reviewed by other authors (EB, ER, MEM) and consensus was achieved through team discussions.

The results of our comparative data analysis are presented in tables, with direct excerpts from the guidelines and policies provided where possible. Two codes (groups and sources of vulnerability, and implications) included more data than others and thus the text has been condensed (i.e. direct citations are not provided). To ensure fidelity to the data, one author (DBR) condensed this text and another (ER) reviewed it to ensure accurate representation of the guidelines and policies.

### Intra-policy holistic analysis

After the inter-policy comparative analysis, we examined the conceptualisation and operationalisation of vulnerability within each policy. Building on the structure developed in our comparative analysis, we assessed the logical consistency between the four content areas of vulnerability. More specifically, we analyzed each policy and guideline in isolation to examine (1) which major content areas are lacking, (2) whether the four content areas (definitions, justifications, groups and sources, and implications) are consistent (e.g. in their meaning) with one another, and (3) what overall impression a guideline or policy user might have about the concept of vulnerability within the document.

## Results

### Inter-policy comparative analysis

#### Defining vulnerability

All policies in our sample reference vulnerability and/or vulnerable subjects, but only three out of eleven explicitly define these terms (Table 2). Of these, the Council for International Organizations of Medical Sciences (CIOMS) and the Tri-Council Policy Statement: Ethical Conduct for Research Involving Humans (TCPS2) guidelines define vulnerability itself, while the International Conference on Harmonisation, Good Clinical Practice (ICH GCP) instead provides a definition of vulnerable subjects. These definitions share similar structures, all defining vulnerability or vulnerable subjects and identifying paradigmatic sources (or causes) of vulnerability. The ICH GCP definition focuses on issues of consent, where a lack of voluntariness in a subject’s decision to participate establishes their vulnerability. The CIOMS and TCPS2 guidelines employ broader language, both stating that vulnerability arises from a subject’s lack of ability to protect their own interests. Both identify sources of vulnerability located within the subject (e.g. a lack of decision-making capacity) and in their environment (e.g. a lack of access to medical care). Only the definition provided by the TCPS2 makes explicit reference to another central ethical concept – that of autonomy. This reference suggests an important link between vulnerability and autonomy, though this connection is not further explained.

**Table 2:** Content regarding definitions of vulnerability and detailing the use of qualifying language

	Policy/Guideline	Explicit definition of vulnerability or vulnerable subjects	Use of qualifying language^a^
Intl			
Intl	Declaration of Helsinki	–	• Some groups and individuals are “*particularly vulnerable*” ([17], Art. 19)
	CIOMS	“*‘Vulnerability’ refers to a substantial incapacity to protect one’s own interests owing to such impediments as lack of capability to give informed consent, lack of alternative means of obtaining medical care or other expensive necessities, or being a junior or subordinate member of a hierarchical group*” ([18], p. 18)	• Persons with serious, potentially disabling or life-threatening diseases are “*highly vulnerable*” ([18], p. 65) • Selection of the “*least vulnerable*” subjects required for research ([18], p. 18)
	UNESCO Declaration	–	• Certain individuals and groups are of “*special vulnerability*” ([19], Art. 8)
EU	Clinical Trials Directive	–	–
	Clinical Trials Regulation	–	–
US, EU, JP, AUS, CA	ICH GCP	Glossary defines vulnerable subjects as “[i]*ndividuals whose willingness to volunteer in a clinical trial may be unduly influenced by the expectation, whether justified or not, of benefits associated with participation, or of a retaliatory response from senior members of a hierarchy in case of refusal to participate*” ([23], Art. 1.61)	–
National			
AUS	National Statement	–	• Where “*potential participants* [in dependent or unequal relationships] *are especially vulnerable*” special measures may be required ([23], p. 53) • Neonates in intensive care have a “*unique developmental vulnerability*” ([23], p. 56) • People with a cognitive impairment, intellectual disability, or mental illness have “*distinctive vulnerabilities as research participants*” and are “*more-than-usually vulnerable to various forms of discomfort or stress*” ([23], p. 58)
CA	TCPS2	“*Vulnerability – A diminished ability to fully safeguard one’s own interests in the context of a specific research project. This may be caused by limited decision-making capacity or limited access to social goods, such as rights, opportunities and power. Individuals or groups may experience vulnerability to different degrees and at different times, depending on their circumstances. See also ‘Autonomy’*” ([24], p. 210)	• Participants, researchers, and research ethics board members may be rendered “*more vulnerable*” during publicly declared emergencies ([24], p. 90) • “*The least organisationally developed communities are the most vulnerable to exploitation*” ([24], p. 130) • Participants may be “*in highly vulnerable circumstances*” because of social or legal stigmatisation ([24], p. 141)
UK	Research Governance Framework	–	–
US	Belmont Report	–	• “*Also, inducements that would ordinarily be acceptable may become undue influences if the subject is especially vulnerable*” ([26], Part C.1)
	Common Rule	–	–

^a^Qualifying language captures nuances about degrees or types of vulnerability

*Intl* international, *EU* European Union, *AUS* Australia, *CA* Canada, *JP* Japan, *UK* United Kingdom, *US* United States, *CIOMS* Council for International Organizations of Medical Sciences, *TCPS2* Tri-Council Policy Statement: Ethical Conduct for Research Involving Humans, *ICH GCP* International Conference on Harmonisation, Good Clinical Practice

The definition provided by the TCPS2 is distinct from the others because it explicitly states that vulnerability is context-dependent, and is experienced “*to different degrees and at different times, depending on* [an individual’s or group's] *circumstances*” ([24], p. 210) . However, qualifying language employed in other policies implicitly suggests a similar view that vulnerability exists on a spectrum or as a matter of degree (Table 2). The Declaration of Helsinki, Australian National Statement, and Belmont Report, for example, discuss participants who are “*particularly vulnerable*” [17], “*more-than-usually vulnerable*” [23], or “*especially vulnerable*” [26], respectively. Unlike the TCPS2, no other guidelines in our sample state explicitly that vulnerability should be thought of as existing on a spectrum, or as a feature that can vary between circumstances.

#### Ethical justifications for the concept of vulnerability

Many guidelines and policies (CIOMS, UNESCO Declaration, Declaration of Helsinki, Australian National Statement, TCPS2, Belmont Report) provide explicit ethical argumentation relating to vulnerability and/or vulnerable subjects. There is significant overlap across the sample between the principles from which obligations or considerations relating to vulnerability arise (see Table 3 for an overview). In all cases where guiding ethical principles are provided by a policy or guideline, vulnerability-related concerns are discussed in the application of each principle.

**Table 3:** Content on the ethical justification of vulnerability and its normative status in each policy

	Policy/Guideline	Justification for vulnerability	Normative status of vulnerability
Intl			
Intl	ICH GCP	–	Consideration for ethics review
	CIOMS	The protection of dependent or vulnerable persons and populations is described itself as a principle; additionally, concerns relating to vulnerability are grounded in both the principles of respect for persons and justice	Fundamental principle/application of other principles
	UNESCO Declaration	Respect for human vulnerability and personal integrity is itself a fundamental principle in this framework	Fundamental principle
	Declaration of Helsinki	Concerns related to vulnerability are themselves principles in this framework	Fundamental principle
EU	Clinical Trials Directive	–	Consideration for ethics review
	Clinical Trials Regulation	–	Consideration for ethics review
National			
AUS	Australian National Statement	Considerations related to vulnerability are discussed in relation to the principles of principles of respect for persons, research merit and integrity, justice, and beneficence	Application of other principles
CA	TCPS2	The principles of respect for persons, justice (fairness and equity), and concern for welfare all entail special obligations regarding vulnerability	Application of other principles
UK	Research Governance Framework	–	Consideration for ethics review
US	Belmont Report	The principles of respect for persons, beneficence, and justice all entail special obligations relating to vulnerability	Application of other principles
	Common Rule	–	Consideration for ethics review

*Intl* international, *EU* European Union, *AUS* Australia, *CA* Canada, *JP* Japan, *UK* United Kingdom, *US* United States, *CIOMS* Council for International Organizations of Medical Sciences, *TCPS2* Tri-Council Policy Statement: Ethical Conduct for Research Involving Humans, *ICH GCP* International Conference on Harmonisation, Good Clinical Practice

The normative status of the concept of vulnerability is inconsistent across policies and guidelines. In certain cases (CIOMS, Australian National Statement, TCPS2, Belmont Report), obligations towards vulnerable research participants arise from the application of other fundamental principles. For example, in the TCPS2, obligations towards those in circumstances of vulnerability are entailed by the policy’s core principles of Respect for Persons, Concern for Welfare, and Justice. In others, concerns or obligations related to vulnerability are themselves characterised as fundamental principles. Specifically, principles 19 and 20 of the Declaration of Helsinki focus on vulnerability, with 19 stating that “[s]*ome groups and individuals are particularly vulnerable and may have an increased likelihood of being wronged or of incurring additional harm*” [17]. Similarly, Article 8 of the UNESCO Declaration promotes “[r]*espect for human vulnerability and personal integrity*” [19] as a principle in and of itself.

The CIOMS guidelines are a unique case in our sample because they characterise vulnerability as both a principle and as a consideration derived from other principles. In the introduction to the CIOMS guidelines, issues of human rights are described as relating to two principles, one of which is the “*protection of dependent or vulnerable persons and populations*” ([18], p. 11), while the principle of Respect for Persons is described as entailing “*at least two fundamental ethical considerations*”, including “*protection of persons with impaired or diminished autonomy, which requires that those who are dependent or vulnerable be afforded security against harm or abuse*” ([18], p. 17).

In the remaining guidelines (ICH GCP, EU Clinical Trials Directive, EU Clinical Trials Regulation, United Kingdom Research Governance Framework, Common Rule), vulnerability is not explicitly discussed in relation to any ethical principles, nor is it described as a guiding principle itself. In these cases, concerns relating to vulnerable persons seem to serve the role of consideration for ethics review or ethical research with no explicit ethical status.

#### Identifying vulnerable groups and individuals

All guidelines and policies in the sample provide means through which vulnerability can be identified. The majority identify subject groups who are likely to be vulnerable. Vulnerable groups identified in our sample are captured in Table 4, along with the corresponding explanations of why a subject group is considered vulnerable or what they are vulnerable to, when these details are available. Notably, while the EU Clinical Trials Directive and Clinical Trials Regulation, as well as the United Kingdom Research Governance Framework, all identify vulnerable subject groups, none of these policies provide any supporting explanation. Further, only four policies (CIOMS, Australian National Statement, TCPS2, and the Common Rule) provide any explanations of what certain identified groups are vulnerable to.

**Table 4:** Vulnerable groups identified in our sample, as well as explanations for this designation, where available

Vulnerable Group (Mentioned in)	Explanation
Grouped by social status or situation	
Prisoners (CIOMS, ICH GCP, Aus. National Statement, TCPS2, Common Rule)	Vulnerable because: • Historically considered vulnerable and “have, at times, been treated unfairly and inequitably in research, or have been excluded from research opportunities”^a^ ([35], p. 8) • Explanation unclear [18, 33] Vulnerable to: • Coercion or undue influence [38]
Certain ethnic, racial minority, or ethnocultural groups (CIOMS, ICH GCP, TCPS2, Belmont Report)	Vulnerable because: • Historically considered vulnerable and “have, at times, been treated unfairly and inequitably in research, or have been excluded from research opportunities”^a^ ([35], p. 8) • May continually be sought as research subjects due to ready availability and administrative convenience; have a dependent status and, frequently, compromised capacity for free consent; are easy to manipulate as a result of their illness or socioeconomic condition ^b^[37] • Explanation unclear [29, 33]
Patients in emergency settings, prospective participants for emergency research (CIOMS, Clinical Trials Regulation, ICH GCP, TCPS2)	Vulnerable because: • Their incapacity to make decisions creates vulnerable circumstances [35] • No explanation [32] • Explanation unclear [29, 33]
Subordinate members of hierarchies or relationships^c^ (CIOMS, ICH GCP, Aus. National Statement)	Vulnerable because: • Voluntary consent may be compromised by expectations of benefit or repercussions from superiors [29, 33] • Pre-existing relationships may compromise the voluntariness of consent because they typically involve unequal status, where one party has influence or authority over the other [34] Vulnerable to: • Being over-researched [29, 34]
Economically disadvantaged persons (Belmont Report, Common Rule)	Vulnerable because: • Dependent status, impaired capacity to consent, easy to manipulate as a result of their illness [37] Vulnerable to: • Coercion or undue influence [38]
Homeless persons (CIOMS, ICH GCP)	• Explanation unclear [29, 33]
Institutionalized persons (TCPS2, Belmont Report)	Vulnerable because: • Historically considered vulnerable and “have, at times, been treated unfairly and inequitably in research, or have been excluded from research opportunities”^a^ ([35], p. 8) • Their ability to fully safeguard their own interests in research may be limited, and their situation may compromise the voluntariness of consent in other ways [35] • May continually be sought as research subjects due to ready availability and administrative convenience; have a dependent status and, frequently, compromised capacity for free consent; are easy to manipulate as a result of their illness or socioeconomic condition^b^ [37]
Nomads (CIOMS, ICH GCP)	• Explanation unclear [29, 33]
Persons in nursing homes (CIOMS, ICH GCP)	• Explanation unclear [29, 33]
Persons lacking political or social power (CIOMS)	• Explanation unclear [29]
Refugees or displaced persons (CIOMS, ICH GCP)	• Explanation unclear [29, 33]
Women (CIOMS, TCPS2)	Vulnerable to: • In some parts of the world, they may be vulnerable to neglect or harm in research “because of their social conditioning to submit to authority, to ask no questions, and to tolerate pain and suffering” ([29], p. 73) Vulnerable because: • Historically considered vulnerable and “have, at times, been treated unfairly and inequitably in research, or have been excluded from research opportunities”^a^ ([35], p. 8)
Countries or communities with limited resources (CIOMS)	Vulnerable to: • Exploitation by sponsors and investigators who are relatively wealthy [29]
Educationally disadvantaged persons (Common Rule)	Vulnerable to: • Coercion or undue influence [38]
Members of communities unfamiliar with modern medical concepts (CIOMS)	• Explanation unclear [29]
Neonates in intensive care (Aus. National Statement)	Vulnerable because: • Developmental vulnerability (potential for long-range impacts on health and development) [34]
Patients in terminal care (Aus. National Statement)	Vulnerable to: • Unrealistic expectations of benefit [34]
Participants and researchers in research that uncovers illegal activities (Aus. National Statement)	Vulnerable because: • Vulnerability may arise because of discovery of participants’ illegal activity [34]
Those with diminished capacity for self-determination (TCPS2)	• Historically vulnerable and “have, at times, been treated unfairly and inequitably in research, or have been excluded from research opportunities”^a^ ([35], p. 8)
The least organizationally developed communities (TCPS2)	Vulnerable to: • Exploitation [35]
Grouped by patient/participant condition	
Children, minors, or young people (CIOMS, Clinical Trials Directive, Clinical Trials Regulation, Aus. National Statement, TCPS2, Common Rule)	Vulnerable because: • Limited freedom or capacity to consent [29, 35] • Vulnerability arising from developmental stage [35] • No explanation [31, 32] • Explanation unclear [34] Vulnerable to: • Coercion or undue influence [38]
Persons with mental illness or mental health problems (Clinical Trials Regulation, Aus. National Statement, TCPS2, UK Research Governance Framework)	Vulnerable because: • Historically considered vulnerable and “have, at times, been treated unfairly and inequitably in research, or have been excluded from research opportunities”^a^ ([35], p. 8) • Unclear [32, 36] Vulnerable to: • Various forms of discomfort and stress [34]
Elderly persons (CIOMS, Clinical Trials Regulation, TCPS2)	Vulnerable because: • Likely to acquire “vulnerability-defining” traits (e.g., institutionalization, dementia) ([29], p. 65] • Historically considered a group in vulnerable circumstances “have, at times, been treated unfairly and inequitably in research, or have been excluded from research opportunities”^a^ ([35], p. 8) • No explanation [32]
Persons with limited (or no) freedom or capacity to consent (CIOMS, Clinical Trials Regulation, ICH GCP)	Vulnerable because: • Relatively (or absolutely) incapable of protecting their own interests [29] • No explanation [32] • Explanation unclear [33] Vulnerable to: • Exploitation for financial gain by guardians [29]
Pregnant or breastfeeding women (Clinical Trials Regulation, Common Rule)	Vulnerable to: • Coercion or undue influence [38] • No explanation [32]
Adults with learning difficulties (UK Research Governance Framework)	• No explanation [36]
Handicapped persons (Common Rule)	• No explanation [38]
Mentally disabled persons (Common Rule)	Vulnerable to: • Coercion or undue influence [38]
Persons who have serious, potentially disabling or life-threatening diseases (CIOMS)	Vulnerable because: • May be treated with drugs or other therapies with unproven safety and efficacy [29]
Very sick persons (Belmont Report)	Vulnerable because: • May continually be sought as research subjects due to ready availability and administrative convenience; have a dependent status and, frequently, compromised capacity for free consent; are easy to manipulate as a result of their illness or socioeconomic condition^b^ [37]
People suffering from multiple chronic conditions (Clinical Trials Regulation)	• No explanation [32]
Persons with a cognitive impairment or intellectual disability (Aus. National Statement)	Vulnerable to: • Various forms of discomfort and stress [34]

^a^It is not clear whether the TCPS2 intends these groups it refers to as having been historically in vulnerable circumstances as still at risk of this. Given that this is mentioned but not negated, we included these groups in our table

^b^The Belmont Report lists a number of vulnerable groups and a series of explanations of their vulnerability. It is unclear whether certain groups were intended to be linked to certain explanations, so all have been included

^c^Within this category, specific subject groups are provided as examples. For the CIOMS these are “medical and nursing students, subordinate hospital and laboratory personnel, employees of pharmaceutical companies, and members of the armed forces or police” ([18, p. 65). The ICH GCP adds pharmacy and dental students and persons kept in detention to this list [18]. The Australian National Statement lists “carers and people with chronic conditions or disabilities, including long-term hospital patients, involuntary patients, or people in residential care or supported acumination; health care professionals and their patients or clients; teachers and their students; prison authorities and prisoners; governmental authorities and refugees; employers or supervisors and employees (including members of the Police and Defence forces); service-providers (government or private) and especially vulnerable communities to whom the service is provided” ([23], p. 53).

The table is grouped by category, and organized by the number of times a group is mentioned in the policies and guidelines

Across the sample, a great number of groups are identified as vulnerable. Counting only those broad groups identified in our table (i.e. excluding the examples of subgroups discussed in the footnote to Table 4), 32 groups were identified; when these subgroups are included, the total number of groups identified as vulnerable expands to 51. Groups most frequently identified are children, minors or young people (discussed in seven policies), prisoners (discussed in five policies), as well as persons with mental health issues, patients in emergency settings, and certain ethnocultural, racial or ethnic minority groups (each discussed in four policies). Concerns for the vulnerability of children centre around consent, with both the CIOMS and TCPS2 guidelines positing a vulnerability arising from their limited freedom or capacity to consent and the Common Rule emphasising children’s vulnerability to coercion or undue influence. The Australian National Statement similarly positions the vulnerability of young people relative to capacity and consent, though it is unclear how this policy conceives of the relationship between these concepts. It outlines various scenarios regarding the vulnerability of young people: in some cases, young people may be able to understand information but their “*relative immaturity means they remain vulnerable*” ([23], p. 50); in other cases they may be “*mature enough to understand and consent* [though] *not vulnerable through immaturity in ways that warrant additional consent*” ([23], p. 50); and in yet other cases, young people may be “*mature enough to understand the relevant information and to give consent, although vulnerable because of immaturity in other respects*” ([23], p. 50). The ‘other respects’ in which immaturity can render young people vulnerable are not made explicit, leaving the designation of vulnerability open to interpretation in this case. Other policies employ similarly open-ended strategies, the CIOMS guidelines most explicitly by listing vulnerable groups and sources of vulnerability, and adding that “[t]*o the extent that these and other classes of people have attributes resembling those of classes identified as vulnerable, the need for special protection of their rights and welfare should be reviewed and applied, where relevant*” ([18], p. 65).

There is little overlap between the explanations provided by policies and guidelines for other frequently-identified vulnerable groups, and there was a lack of explanation from at least two of them for prisoners, patients in emergency settings, and ethnocultural and racial minorities. For over half of the groups identified across our sample, an explanation of their vulnerability was unclear or lacking entirely. The EU Clinical Trials Directive and Clinical Trials Regulation and United Kingdom Research Governance Framework provide no explanation or justification for any of the groups they designate as vulnerable, and while the Common Rule specifies that it is concerned with vulnerability to coercion or undue influence, it does not address 'handicapped persons' in this explanation despite also identifying them as a vulnerable subject group. The CIOMS and ICH GCP guidelines, on the other hand, provide definitions of vulnerable subjects and explanations for some vulnerable groups. However, both of these policies include categories of 'other vulnerable groups' and fail to provide any connection between these other groups and their overarching definition of vulnerability. As such, it is unclear whether they are designated as vulnerable on some other unstated grounds.

Some policies and guidelines identify sources or circumstances of vulnerability independently, i.e. without any relation or association to a specific vulnerable group. For example, neither the Declaration of Helsinki nor the UNESCO Declaration identifies any particular subject groups as vulnerable. Instead, they identify characteristics of vulnerable participants or key sources of vulnerability (Table 5). It is important to note that, while the TCPS2 does identify certain groups as likely to be in vulnerable circumstances, it qualifies any such labels, emphasising that “[i]*ndividuals should not automatically be considered vulnerable simply because of assumptions made about the vulnerability of the group to which they belong*” ([24], p. 54).

**Table 5:** Sources of vulnerability identified independently from vulnerable groups

Policy/Guideline	Sources of vulnerability
Declaration of Helsinki	An increased likelihood of being wronged or of incurring harm
UNESCO Declaration	Persons may be rendered vulnerable by disease or disability or other personal, societal or environmental conditions
TCPS2	Persons may be in vulnerable circumstances because of social or legal stigmatisation associated with their activity or identity

*TCPS2* Tri-Council Policy Statement: Ethical Conduct for Research Involving Humans

#### Implications of vulnerability in research

All policies in our sample identify practical implications of vulnerability in research, i.e. responses to vulnerability in the design and review of research and to vulnerable participants themselves. A wide range of implications were identified, some directed explicitly towards REBs and/or investigators but the majority formulated more broadly with no specific group targeted. Further, these implications span the research process, from considerations important in the design of research to actions that must be taken when vulnerable persons are participating in research (Table 6).

**Table 6:** Implications of vulnerability, grouped by theme

*Restrictions for research with vulnerable groups or individuals*	*Policy/Guideline*
When research is carried out with vulnerable participants it should be responsive to the needs, conditions, or priorities of the vulnerable group involved	Declaration of Helsinki; CIOMS
Vulnerable subjects should be involved in research only when it cannot be carried out with less vulnerable subjects	CIOMS
Special justification is required for involving vulnerable groups in research and appropriateness ought to be demonstrated	CIOMS; Belmont Report
Children should not be included in early-phase research until therapeutic effects have been shown in adults	CIOMS
Opportunities to participate in and influence research affecting their welfare should not be withheld from vulnerable groups	TCPS2
Members of vulnerable groups are entitled to access the benefits of research	CIOMS
Children must be involved in studies of medicinal products likely to be of value to them	EU Clinical Trials Directive
People with a cognitive impairment, intellectual disability, or mental illness are entitled to participate in research, which need not be limited to their particular impairment, disability, or illness	Australian National Statement
Research with communities vulnerable to exploitation should strive to enhance capacity for participation	TCPS2
Patients receiving high-risk clinical care should not be inappropriately included in or excluded from research	TCPS2
Risk to vulnerable subjects is justified when it arises from interventions that will provide a direct health benefit, or when it will benefit the subject’s population group	CIOMS
*Special protections and obligations*	
Individuals and groups of special vulnerability should be protected	UNESCO Declaration
Special ethical obligations exist towards vulnerable subjects	TCPS2
Vulnerable subjects should receive special/specific protections	Declaration of Helsinki
Groups or individuals in vulnerable circumstances may need or desire special measures to ensure their safety in a specific research project	TCPS2
Vulnerable subjects should be afforded security against harm or abuse	CIOMS
Special (or additional) protections for the rights and welfare of vulnerable subjects should be applied	CIOMS; Common Rule
*Attention and consideration*	
Special attention should be paid to trials involving vulnerable subjects	ICH GCP
Special attention or regard should be paid to vulnerable communities, groups, or persons	UNESCO Declaration; TCPS2
Researchers and REBs should recognise and address changes in participants’ circumstances that may impact their vulnerability	TCPS2
*Research ethics board composition*	
REBs reviewing research with vulnerable subjects should include members with expertise on these populations	Common Rule; EU Clinical Trials Regulation
Community members on REBs ought to reflect participant’s perspectives, particularly important when participants are vulnerable and/or risks are high	TCPS2
*Assessing harms, risks and benefits*	
For those gauging the severity of harm in research, the vulnerability of a population will be relevant	Australian National Statement
The existence of vulnerable circumstances may require greater effort to minimise risks/maximise benefits to participants	TCPS2
Care must be taken to ensure the risks and burdens of proposed research with persons with a cognitive impairment, intellectual disability, or mental illness are justified by potential benefits	Australian National Statement
*Recruitment practices*	
The vulnerability of persons in unequal, dependent relationships must be taken into account when considering recruiting these persons	National Statement
*Process of informed consent*	
Consent may need to be re-confirmed in research where participants are vulnerable	National Statement
The method of consent in qualitative research depends, in part, on the vulnerability of the research participant; the method must be tailored for their protection	National Statement; TCPS2
When requirements of free, informed, ongoing consent cannot be met, vulnerable participants ought to be involved in decision-making, i.e. obtaining assent, asking about their feelings regarding participation	TCPS2
Clinician-researchers must take care not to overplay the benefits of research participation to vulnerable patients, who may be misled to enter research with false hope	TCPS2
Inducements that may not be excessive or inappropriate for other participants may be undue influences if the subject is especially vulnerable	Belmont Report
Care should be taken in the informed consent process to ensure that women vulnerable to coercion have adequate time and a proper environment in which to take decisions	CIOMS
Care should be taken in the informed consent process for adults with mental health problems or learning difficulties to ensure that information is provided in the appropriate format and that the roles and responsibilities of those involved are clearly explained and understood	UK Research Governance Framework
Additional consent from a parent or guardian may be required for young people who are vulnerable through immaturity in ways that warrant this	National Statement
Researchers should invite participants in dependent or unequal relationships to discuss their participation with someone who can support them in making their decision; especially vulnerable participants in these circumstances should be offered participant advocates	National Statement
*Debriefing*	
REBs must assess risks and benefits of debriefing participants and whether debriefing plan is appropriate for participants, especially when they are vulnerable	TCPS2

*REB* research ethics board, *CIOMS* Council for International Organizations of Medical Sciences, *TCPS2* Tri-Council Policy Statement: Ethical Conduct for Research Involving Humans, *ICH GCP* International Conference on Harmonisation, Good Clinical Practice

A majority of policies and guidelines identify implications relating to restrictions for research with vulnerable groups or individuals, but these entail both negative and positive duties. Overall, these policies and guidelines propose that the involvement of vulnerable groups in research ought to be restricted to some extent; vulnerable persons ought to be involved only when the research cannot be carried out with persons who are less vulnerable and special justification is required for their involvement. However, when these persons are involved in research, additional actions are required, such as the design of research that is responsive to their needs or priorities and the provision of benefits relevant to their group/subject population. Across our sample, a common underlying assumption seems to be that vulnerable groups can and should be involved in research, but that additional measures are required to ensure this involvement occurs in an ethical manner. In fact, several policies (CIOMS, EU Clinical Trials Directive, Australian National Statement, and TCPS2) assert that vulnerable groups have a right to participate in research and access its benefits, and while the others do not identify such an entitlement, none go so far as to state that the outright exclusion of vulnerable groups from research best serves to protect them.

The implications of vulnerability all tend towards careful inclusion rather than outright exclusion of vulnerable groups from research. However, there is more variability regarding the extent to which these protections afford agency to vulnerable subjects. The majority specify considerations and actions for researchers and REBs, with few explicitly identifying the desires of these individuals as relevant in the application of these measures. The TCPS2 in particular puts forth numerous measures intended to promote the agency of those in vulnerable circumstances. For example, it is suggested that they should be afforded opportunities to influence research and that research ought to enhance vulnerable persons’ capacity for participation. Furthermore, the TCPS2 guidance states more broadly that vulnerable groups may need or desire special measures to ensure their safety, suggesting a role for participants in the design and implementation of their protections.

In addition to conditions and restrictions for research involvement, the process of informed consent is a major area of focus in the policies and guidelines. Here in particular there is an emphasis on the provision of meaningful support to enable vulnerable persons to offer a fully informed consent to research. Mechanisms of support include ensuring adequate time and an appropriate environment (CIOMS), as well as ensuring that information is fully explained and understood (United Kingdom Research Governance Framework). Additionally, the Australian National Statement uniquely suggested that participants be given the option of using a participant advocate within the consent process.

### Holistic policy analysis

Of the 11 policies and guidelines in our sample, only two, the CIOMS guidelines and TCPS2, meet our criteria for a full conceptualisation of vulnerability, addressing all content areas (Table 7). In this section, we present the results of our intra-policy analysis of vulnerability with a narrative about each policy statement, addressing (1) which major content areas are lacking, (2) whether the content areas are consistent (i.e. in their meaning) with one another, and (3) what overall impression a guideline or policy user might have about the concept of vulnerability within the document.

**Table 7:** Major content areas of vulnerability addressed within each policy/guideline

Policy/Guideline	Definition: What is vulnerability?	Groups/Sources: Who is vulnerable and why?	Justifications: What ethical concern(s) does vulnerability reflect?	Implications: How should we respond to vulnerability in research?
International				
Declaration of Helsinki	–	X	X	X
CIOMS	X	X	X	X
UNESCO Declaration	–	X	X	X
EU Clinical Trials Directive	–	X	–	X
EU Clinical Trials Regulation	–	X	–	X
ICH GCP	X	X	–	X
National				
Australian National Statement	–	X	X	X
TCPS2	X	X	X	X
UK Research Governance Framework	–	X	–	X
Belmont Report	–	X	X	X
Common Rule	–	X	–	X

*CIOMS* Council for International Organizations of Medical Sciences, *TCPS2* Tri-Council Policy Statement: Ethical Conduct for Research Involving Humans, *ICH GCP* International Conference on Harmonisation, Good Clinical Practice

#### International policies and guidelines

##### Declaration of Helsinki

The Declaration conveys a harm/wrong-based conceptualisation of vulnerability that is internally coherent due to its broad language. It does not identify what these wrongs or harms might consist of and, because concern for vulnerability is presented as a fundamental principle, interpretation cannot be guided by other ethical principles. Implications of vulnerability focus on the need for responsive research, special justification for involving vulnerable persons, and to-group benefits, suggesting these harms include the unfair distribution of the risks and benefits of research.

##### CIOMS

These guidelines present an autonomy-based conceptualisation of vulnerability that is comprehensive in scope but lacks internal clarity in its discussion of vulnerable groups. While the provided definition focuses on vulnerability as stemming from an incapacity to protect one’s own interests owing to both individual and environmental features, vulnerability is also explicitly linked to justice-based concerns about the distribution of the risks and benefits of research. The identified implications of vulnerability thus correspond to concerns relating to participants’ ability to provide free and informed consent and relating to the appropriateness of involving vulnerable participants in research. There is a lack of clarity and consistency, however, in the discussion of vulnerable groups. The CIOMS guidelines distinguishes between three types of vulnerable groups – those who are “*conventionally considered vulnerable*”, those who are vulnerable due to social pressures (i.e. persons in dependent relationship with researchers, such as students or pharmaceutical employees), and “*other groups or classes*” ([18], p. 64) for whom no explanation is provided and who do not, on their face, bear a significant resemblance to these other groups.

##### UNESCO Declaration

The Declaration is concerned with both a general “*human vulnerability*” and a more particular “*special vulnerability*” ([19], Art. 8), neither of which are defined. Its identification of personal, societal and environmental conditions as sources of vulnerability suggests a concept with wide-ranging concerns. The Declaration identifies respecting the personal integrity of vulnerable groups as a key implication, suggesting that vulnerability may consist, at least in part, of risks to one’s personal integrity. Since concerns relating to vulnerability are presented as fundamental principles, their interpretation cannot be guided by other ethical principles.

##### EU Clinical Trials Directive

The Clinical Trials Directive conveys a primarily consent-based vulnerability, with children as the focus of its vulnerability-related regulations. The implications it identifies focus on obtaining proxy consent and assent, but also on the need to avoid financial inducements for participation, suggesting a concern for a risk of exploitation. Other implications include a need to perform research with children in which group benefits will be obtained and ensuring the interests of the patient prevail over those of society. As such, in addition to concerns relating to consent, the Directive implicitly relates vulnerability to concerns with the distribution of the benefits and burdens of research. The Directive does not provide an ethics framework, so interpretation of this guidance cannot be guided by ethical principles.

##### EU Clinical Trials Regulation

The Clinical Trials Regulation conveys a mixed concept of vulnerability, concerned both with issues of consent and increased health risks. While vulnerability is not defined and no explanation for the vulnerability of listed groups is provided, they can be grouped by those assumed to face issues of consent in research (people affected by mental health disorders, minors, and incapacitated subjects) and those who may be at greater physical (i.e. health) risks in research (frail or older people, people suffering from chronic conditions, and pregnant or breastfeeding women). The implications identified do not fall along this consent/health risk distinction, however, with a need for research to improve treatments a key implication for frail or older people, people with chronic conditions, and people affected by mental health disorders, and the need for special expertise in research ethics review identified as a specific consideration for minors, incapacitated subjects, and pregnant or breastfeeding women. No ethical framework is provided in the Regulation to facilitate interpretation of this guidance.

##### ICH GCP

These guidelines present a consent-based concept of vulnerability that lacks internal clarity due to its broad scope of vulnerable groups. Vulnerable subjects are defined as those whose ability to provide voluntary consent may be compromised by social pressures, and the first category of groups listed is clearly linked to this definition. However, it is not clear how the wide range of 'other vulnerable groups' relates to this definition or which characteristics are thought to render them vulnerable. The guidelines do not provide an ethical framework to facilitate interpretation of the concept of vulnerability.

#### National policies and guidelines

##### Australian National Statement

The National Statement suggests a comprehensive conceptualisation of vulnerability relating to concerns about consent, fair involvement in research, and a balance of risks and benefits to participants. It favours a group-specific approach to vulnerability, where this concept is discussed largely in reference to specific groups. General statements about vulnerability suggest that it is an important factor when considering the appropriate method of consent. While vulnerability is not defined, explanations for the vulnerability of all identified groups are provided and are discussed with reference to the Statement’s guiding ethical principles. Interestingly, explanations of the vulnerability of identified groups the principles from which obligations to those groups stem do not always line up. In some cases, the relationship is clear; the vulnerability of young persons originates in their lack of ability to provide consent and is linked to respect for persons, and the vulnerability of neonates in intensive care originates in the risks of long-term harms and is linked to beneficence. However, while persons in pre-existing/dependent relationships with researchers are said to face issues providing voluntary consent, the key implication relating to this group is grounded in the principle of justice (i.e. ensuring they are not over-researched). Similarly, while persons with terminal illness are said to be vulnerable to unrealistic expectations of benefit (i.e. may have a compromised ability to consent), the key response to this is to balance the benefits and burdens of research and is grounded in beneficence.

#### TCPS2

The TCPS2 presents an autonomy-based conceptualisation of vulnerability that is comprehensive in scope. The provided definition of vulnerability states that it stems from a diminished ability to protect one’s own interests caused by both individual (e.g. lack of decision-making capacity) and environmental (e.g. lack of access to social goods) factors. Importantly, vulnerability is said to be context-specific and dynamic, discouraging assumptions of vulnerability based on group membership. However, the policy still relies on the identification of groups likely to be vulnerable, as well as the identification of circumstances that can create vulnerability for a participant. While the definition of vulnerability itself is implicitly linked to the principle of autonomy, obligations towards participants in vulnerable circumstances are more comprehensive and are grounded in the principles of respect for persons, concern for welfare and justice.

##### United Kingdom Research Governance Framework

The framework conveys a consent-based conceptualisation of vulnerability that is narrow in scope, labelling adults who may have issues with understanding and decision-making as vulnerable. Consistent with this, the implications of vulnerability focus on providing participants with the necessary support in the informed consent process. Since no ethical framework or principles are discussed relative to vulnerability, these cannot be used to facilitate interpretation of the guidance.

##### Belmont Report

The Report conveys a consent-based conceptualisation of vulnerability that lacks clarity in the features of vulnerability it aims to target. It is assumed that vulnerable subjects have a “*dependent status and frequently compromised capacity for free consent*” ([26], Part C. 3), which seems to form the basis of their vulnerability. Special considerations about vulnerable subjects are discussed in reference to respect for persons (ordinary inducements may be come undue influences for vulnerable populations), beneficence (special justification is required for research with vulnerable subjects) and justice (vulnerable subjects must be protected from over-recruitment to research).

##### Common Rule

The Common Rule conveys a consent-based conceptualisation of vulnerability that lacks internal clarity regarding its scope. A number of groups are identified as vulnerable, including handicapped persons, but while the other groups are said to be vulnerable to coercion or undue influence, no explanation is provided for handicapped persons. Similarly, the implications of vulnerability include concern for equitable subject selection and the provision of additional safeguards, but handicapped persons are never associated with these protections. Without a definition of vulnerability, it is not clear what special vulnerability handicapped persons may be faced with in research.

## Discussion

The objective of this analysis was to describe the concept of vulnerability in research ethics policies and guidelines, and to assess how it is conceptualised and operationalised. All policies and guidelines employed the concept of vulnerability but very few define it. Instead, vulnerability is most frequently discussed in terms of vulnerable groups, with some attention given to the sources of vulnerability, and the implications of conducting research with vulnerable participants. In many respects the policies come out, on the whole, as richer and more complex than some scholarly analyses of the concept of vulnerability suggest [6, 28, 29]. For example, the policies and guidelines identify sources of vulnerability that are both individual and situational [29]; vulnerability can stem from a lack of capacity or from one’s health status, but also from social pressures that may impact one’s ability to make a free and informed decision, consistent with some scholarly perspectives [30]. Responding to vulnerability requires caution and special consideration on the part of researchers and REBs but, ultimately, the implications identified in our study suggest that participant vulnerability need not signal a need for exclusion from research. The few explicit definitions in our sample define vulnerability as a deficiency of the participant, as an inability to protect one’s interests in research. The majority of other guidelines and policies implicitly convey a similar conceptualisation of vulnerability as a deficiency in a participant’s ability to provide voluntary informed consent. Accordingly, even though there is some diversity and richness in policies, it tends to be scattered across multiple policies and relies on implicit assumptions about the definition and nature of vulnerability. Indeed, a significant analytic effort was required to bring structure to the data and yield the guidance captured in this paper. We further discuss how our findings relate to (1) previous critiques found in the scholarly literature and (2) the role of stakeholder engagement in the process of refining the concept of vulnerability in research ethics policies and guidance.

### Previous critiques from the scholarly literature

Within the scholarly literature several critiques of vulnerability in research ethics guidelines have been voiced. First, concerns have been raised that the manner in which vulnerability is defined and operationalised in research ethics governance stereotypes and reinforces stigma about whole categories of individuals [9, 12, 31]. Our results reinforce these concerns, as the reliance on listing groups of vulnerable persons is rampant. This labelling [6] or sub-population [30] approach does little to bring attention to the importance of context and of assessing the characteristics of individual research participants beyond their membership in a group [5, 6, 9]. It is important to note that research protocols create groups through sampling “*regardless of whether the sample is drawn from a naturally occurring community*” ([9], p. 2221). Understanding this point underscores the fact that group membership in this context may not well capture the various relevant aspects (and potential vulnerabilities) in an individual participant’s situation. This may result in inappropriate and ineffective protections being applied in some protocols. Group listings may also cause confusion due to the broadness of some labels (e.g. persons with mental illness or mental health problems). Furthermore, it seems that the designation of some groups as vulnerable may be based on assumptions not supported by evidence (e.g. the designation of pregnant women as vulnerable to coercion or undue influence in the Common Rule).

Another major concern has been that vulnerability, as conceived of in the guidelines, focuses overwhelmingly on a lack of ability to consent [10], blinding researchers and REBs to other relevant types of vulnerability, relating, for example, to an increased risk of exploitation [32] or a lack of basic rights [33]. While vulnerability is rarely defined, the majority of policies and guidelines convey implicitly that vulnerability is fundamentally an inability to provide free and informed consent. However, the implications of vulnerability often move beyond consent, addressing issues of fair subject selection and favourable risk benefit assessments. In addition to providing explicit definitions for what, exactly, is meant when the term ‘vulnerability’ is used, the clarity and usability of policies and guidelines could be improved by ensuring that these definitions clearly relate to the concerns with which vulnerability is associated.

Though they recognise both individual and contextual sources of vulnerability, all policies and guidelines conveyed that vulnerability is a personal characteristic. Even the TCPS2, with its notable emphasis on vulnerability as a context-dependent feature, ultimately defines it as a person’s inability to protect their own interests in research. In contrast, a growing body of scholarly literature converges around the notion that vulnerability is a relational feature, borne of power asymmetries between participants and research staff, investigators and institutions [10, 31, 34]. Adopting such a view in research ethics guidelines may better serve participants, encouraging measures that would empower and promote their agency in the research context [34]. Furthermore, the focus on research participants neglects how research environments (e.g. the existence of conflicts of interest) can actively contribute to disempowering research participants/research subjects and thus create the need for remediation that does not necessarily concern the research participant per se [6, 10].

### A need for evidence and stakeholder engagement to refine research ethics policies and guidance on vulnerability

Research ethics guidelines and policies typically stress the importance of vulnerability. However, it has been argued that vulnerability is not a substantive ethical concept in itself, serving only as a marker of other research ethics concerns already captured by existing concepts such as harm or consent [35]. This is certainly an important conceptual concern, but what may be of greater relevance in the realm of policy development is the degree to which the concept of vulnerability is a useful, effective tool for those designing, reviewing and conducting research [5, 6]. It may be the case that vulnerability merely serves to signal concerns relating to other pre-existing ethical concepts, but if these concerns would be otherwise missed, the concept would then be proven to have a vital practical function in research ethics. A few authors have made explicit claims to that effect. For example, Kipnis argues that vulnerability stems from impairments to one’s ability to provide voluntary informed consent. He identifies six types of vulnerability which all signal potential issues with a participant’s ability to consent: cognitive, deferential and medical vulnerability, all of which relate to characteristics of the participant themselves, and juridic, allocational and infrastructural vulnerability, all of which relate to factors in the participant’s environment [30]. These categories help bring attention to more specific aspects that generate vulnerability. Luna argues that vulnerability, when conceived of as dynamic, flexible and inessential, can serve as “*a fine grain tool to analyze, interpret, and evaluate the research situation*” ([6], p. 130). She proposes that vulnerability be conceived of through the metaphor of layers, in which different layers of vulnerability can operate and interact within a given participant’s circumstances. Luna’s account of vulnerability thus provides researchers and REBs with a conceptual tool with which to examine a research participant’s circumstances, identify potential vulnerabilities (e.g. relating to capacity or social pressure in the consent process), and develop targeted strategies for their remediation.

In spite of these more sophisticated proposals, there is a dearth of empirical evidence on the functioning of research ethics committees and outcomes of research ethics policies and, to our knowledge, few studies have examined the impact or understandings of the concept of vulnerability based on research ethics guidelines and/or more elaborate scholarly accounts. Empirical evidence has shown that an understanding of vulnerability in the context of research cannot be assumed to be universal – in a study with Russian and Romanian research ethics trainees, Loue and Loff [36] found that, at the initiation of their training, their existing understanding of vulnerability varied considerably from conceptualisations in the international guidelines. A study by Sengupta et al. [37] gathered researchers’ perspectives on vulnerability in HIV/AIDS clinical trials and on the Common Rule guidance related to vulnerability and found that researchers assessed vulnerability in relation to situational factors that can render participants vulnerable, and that they emphasised the need to assess vulnerability on a case-by-case basis (i.e. rather than relying on a group-based strategy). Taken together, these studies underscore the need for policymakers to clearly delineate and define the concerns vulnerability is intended to encompass, and to assess the alignment of these views with those of research stakeholders. Further, there is a need to assess the outcomes of vulnerability-related guidance and policy and to understand whether protections are actually effective and their impact on vulnerable participants themselves. For example, there are crucial questions about the actual usability and impact of such guidelines as well as the potential need for mid-level guidance between general guidelines and the actual analyses of REBs [38]. It has been suggested that more elaborate, on-the-ground guidance on vulnerability would be beneficial to help REBs direct their attention to the most pertinent concerns [30]. In the process of developing such guidance, the voices of those concerned by the application of what sometimes appears as a label of vulnerability could be instrumental in moving forward and avoiding the perpetuation of stereotyping or stigmatising accounts of vulnerability [34]. In this endeavour, the perspectives of researchers and REBs, but also of research participants, who seem to have been largely left out of the development of research ethics guidelines, could be investigated.

## Conclusion

Our in-depth analysis of human research ethics guidelines and policies allowed us to analyse different perspectives on the concept of vulnerability, including the definitions, justifications, sources, and implications of vulnerability for researchers and REBs. In some respects, this synthetic account yielded a richer perspective than sometimes admitted in scholarly literature. At the same time, there are conceptual gaps within individual guidelines and policies that require the attention of those charged with their development. This lack of clarity could diminish the usability of the guidance put forth in policies and therefore undermine its impact on research practices. Policymakers should revisit the concept of vulnerability to ensure each of its key components is spelled out, and that these components are internally consistent (i.e. within individual guidelines). Practically-oriented refinement of vulnerability could be facilitated by engaging research stakeholders and examining the concrete impact of guidance and policy related to vulnerability.
