# Erratum to: The concept of ‘vulnerability’ in research ethics: an in-depth analysis of policies and guidelines

**DOI:** 10.1186/s12961-017-0186-8

**Published:** 2017-04-03

**Authors:** Dearbhail Bracken-Roche, Emily Bell, Mary Ellen Macdonald, Eric Racine

**Affiliations:** 1 Neuroethics Research Unit, Institut de recherches cliniques de Montréal, 110 Avenue des Pins Ouest, Montréal, QC H2W 1R7 Canada; 2 Biomedical Ethics Unit and Division of Experimental Medicine, McGill University, Montréal, QC Canada; 3 Faculty of Dentistry, Oral Health and Society Research Unit, McGill University, 2001 McGill College, Suite 500, Montréal, QC H3A 1G1 Canada; 4 Department of Medicine and Department of Social and Preventive Medicine, Université de Montréal, Montréal, QC Canada; 5 Department of Neurology and Neurosurgery, McGill University, Montréal, QC Canada

Unfortunately the refernces within Table 4 of this article [1] were not corrected during published and therefore published incorrectly. Please see Table 4 corrected below.

**Table 4:** Vulnerable groups identified in our sample, as well as explanations for this designation, where available

Vulnerable Group (Mentioned in)	Explanation
Grouped by social status or situation	
Prisoners (CIOMS, ICH GCP, Aus. National Statement, TCPS2, Common Rule)	Vulnerable because: •Historically considered vulnerable and “have, at times, been treated unfairly and inequitably in research, or have been excluded from research opportunities”^a^ ([24], p. 8) •Explanation unclear [18, 22] Vulnerable to: •Coercion or undue influence [27]
Certain ethnic, racial minority, or ethnocultural groups (CIOMS, ICH GCP, TCPS2, Belmont Report)	Vulnerable because: •Historically considered vulnerable and “have, at times, been treated unfairly and inequitably in research, or have been excluded from research opportunities”^a^ ([24], p. 8) •May continually be sought as research subjects due to ready availability and administrative convenience; have a dependent status and, frequently, compromised capacity for free consent; are easy to manipulate as a result of their illness or socioeconomic condition^b^ [26] •Explanation unclear [18, 22]
Patients in emergency settings, prospective participants for emergency research (CIOMS, Clinical Trials Regulation, ICH GCP, TCPS2)	Vulnerable because: •Their incapacity to make decisions creates vulnerable circumstances [24] •No explanation [21] •Explanation unclear [18, 22]
Subordinate members of hierarchies or relationships^c^ (CIOMS, ICH GCP, Aus. National Statement)	Vulnerable because: •Voluntary consent may be compromised by expectations of benefit or repercussions from superiors [18, 22] •Pre-existing relationships may compromise the voluntariness of consent because they typically involve unequal status, where one party has influence or authority over the other [23] Vulnerable to: •Being over-researched [18, 23]
Economically disadvantaged persons (Belmont Report, Common Rule)	Vulnerable because: •Dependent status, impaired capacity to consent, easy to manipulate as a result of their illness [26] Vulnerable to: •Coercion or undue influence [27]
Homeless persons (CIOMS, ICH GCP)	•Explanation unclear [18, 22]
Institutionalized persons (TCPS2, Belmont Report)	Vulnerable because: •Historically considered vulnerable and “have, at times, been treated unfairly and inequitably in research, or have been excluded from research opportunities”^a^ ([24], p. 8) •Their ability to fully safeguard their own interests in research may be limited, and their situation may compromise the voluntariness of consent in other ways [24] •May continually be sought as research subjects due to ready availability and administrative convenience; have a dependent status and, frequently, compromised capacity for free consent; are easy to manipulate as a result of their illness or socioeconomic condition^b^ [26]
Nomads (CIOMS, ICH GCP)	•Explanation unclear [18, 22]
Persons in nursing homes (CIOMS, ICH GCP)	•Explanation unclear [18, 22]
Persons lacking political or social power (CIOMS)	•Explanation unclear [18]
Refugees or displaced persons (CIOMS, ICH GCP)	•Explanation unclear [18, 22]
Women (CIOMS, TCPS2)	Vulnerable to: •In some parts of the world, they may be vulnerable to neglect or harm in research “because of their social conditioning to submit to authority, to ask no questions, and to tolerate pain and suffering” ([18], p. 73) Vulnerable because: •Historically considered vulnerable and “have, at times, been treated unfairly and inequitably in research, or have been excluded from research opportunities”^a^ ([24], p. 8)
Countries or communities with limited resources (CIOMS)	Vulnerable to: •Exploitation by sponsors and investigators who are relatively wealthy [18]
Educationally disadvantaged persons (Common Rule)	Vulnerable to: •Coercion or undue influence [27]
Members of communities unfamiliar with modern medical concepts (CIOMS)	•Explanation unclear [18]
Neonates in intensive care (Aus. National Statement)	Vulnerable because: •Developmental vulnerability (potential for long-range impacts on health and development) [23]
Patients in terminal care (Aus. National Statement)	Vulnerable to: •Unrealistic expectations of benefit [23]
Participants and researchers in research that uncovers illegal activities (Aus. National Statement)	Vulnerable because: •Vulnerability may arise because of discovery of participants’ illegal activity [23]
Those with diminished capacity for self-determination (TCPS2)	•Historically vulnerable and “have, at times, been treated unfairly and inequitably in research, or have been excluded from research opportunities”^a^ ([24], p. 8)
The least organizationally developed communities (TCPS2)	Vulnerable to: •Exploitation [24]
Grouped by patient/ participant condition	
Children, minors, or young people (CIOMS, Clinical Trials Directive, Clinical Trials Regulation, Aus. National Statement, TCPS2, Common Rule)	Vulnerable because: •Limited freedom or capacity to consent [18, 24] •Vulnerability arising from developmental stage [24] •No explanation [20, 21] •Explanation unclear [23] Vulnerable to: •Coercion or undue influence [27]
Persons with mental illness or mental health problems (Clinical Trials Regulation, Aus. National Statement, TCPS2, UK Research Governance Framework)	Vulnerable because: •Historically considered vulnerable and “have, at times, been treated unfairly and inequitably in research, or have been excluded from research opportunities”^a^ ([24], p. 8) •Unclear [21, 25] Vulnerable to: •Various forms of discomfort and stress [23]
Elderly persons (CIOMS, Clinical Trials Regulation, TCPS2)	Vulnerable because: •Likely to acquire “vulnerability-defining” traits (e.g., institutionalization, dementia) ([18], p. 65] •Historically considered a group in vulnerable circumstances “have, at times, been treated unfairly and inequitably in research, or have been excluded from research opportunities”^a^ ([24], p. 8) •No explanation [21]
Persons with limited (or no) freedom or capacity to consent (CIOMS, Clinical Trials Regulation, ICH GCP)	Vulnerable because: •Relatively (or absolutely) incapable of protecting their own interests [18] •No explanation [21] •Explanation unclear [22] Vulnerable to: •Exploitation for financial gain by guardians [18]
Pregnant or breastfeeding women (Clinical Trials Regulation, Common Rule)	Vulnerable to: •Coercion or undue influence [27] •No explanation [21]
Adults with learning difficulties (UK Research Governance Framework)	•No explanation [25]
Handicapped persons (Common Rule)	•No explanation [27]
Mentally disabled persons (Common Rule)	Vulnerable to: •Coercion or undue influence [27]
Persons who have serious, potentially disabling or life-threatening diseases (CIOMS)	Vulnerable because: •May be treated with drugs or other therapies with unproven safety and efficacy [18]
Very sick persons (Belmont Report)	Vulnerable because: •May continually be sought as research subjects due to ready availability and administrative convenience; have a dependent status and, frequently, compromised capacity for free consent; are easy to manipulate as a result of their illness or socioeconomic condition^b^ [26]
People suffering from multiple chronic conditions (Clinical Trials Regulation)	•No explanation [21]
Persons with a cognitive impairment or intellectual disability (Aus. National Statement)	Vulnerable to: •Various forms of discomfort and stress [23]

^a^It is not clear whether the TCPS2 intends these groups it refers to as having been historically in vulnerable circumstances as still at risk of this. Given that this is mentioned but not negated, we included these groups in our table

^b^The Belmont Report lists a number of vulnerable groups and a series of explanations of their vulnerability. It is unclear whether certain groups were intended to be linked to certain explanations, so all have been included

^c^Within this category, specific subject groups are provided as examples. For the CIOMS these are “medical and nursing students, subordinate hospital and laboratory personnel, employees of pharmaceutical companies, and members of the armed forces or police” ([18], p. 65). The ICH GCP adds pharmacy and dental students and persons kept in detention to this list [22]. The Australian National Statement lists “carers and people with chronic conditions or disabilities, including long-term hospital patients, involuntary patients, or people in residential care or supported acumination; health care professionals and their patients or clients; teachers and their students; prison authorities and prisoners; governmental authorities and refugees; employers or supervisors and employees (including members of the Police and Defence forces); service-providers (government or private) and especially vulnerable communities to whom the service is provided” ([23], p. 53)

The table is grouped by category, and organized by the number of times a group is mentioned in the policies and guidelines
